# Training the Next Generation of Researchers to Advance Cancer Health Equity: Five Years of Experience from the Florida-California Cancer Research, Education and Engagement (CaRE^2^) Health Equity Center

**DOI:** 10.1007/s13187-025-02676-1

**Published:** 2025-06-26

**Authors:** John M. Allen, Bereket Mochona, Kinfe Redda, Debra Lyon, Brooke Hensel, John D. Carpten, Mariana C. Stern, Romonia Renee Reams, Folakemi T. Odedina, Diana J. Wilkie, Ite A. Offringa

**Affiliations:** 1 Department of Pharmacy Practice, Purdue University, West Lafayette, IN USA; 2 Florida A&M University, Tallahassee, FL USA; 3 University of Florida, Gainesville, FL USA; 4 University of Florida, Orlando, FL USA; 5 Beckman Research Institute of City of Hope, City of Hope National Cancer Institute, Duarte, CA USA; 6 Department of Population and Public Health Sciences, USC Norris Comprehensive Cancer Center, Keck School of Medicine, University of Southern California, Los Angeles, CA USA; 7 Mayo Clinic, Jacksonville, FL USA; 8 Keck School of Medicine, University of Southern California, Los Angeles, CA USA

**Keywords:** Research and education, Training program, Cancer health disparities, Unrepresented minorities

## Abstract

The underrepresentation of racial and ethnic minority scientists in research is a significant barrier to eliminating cancer health disparities. There is a compelling need to develop a cadre of racially and ethnically diverse, well-trained scientists to effectively meet the nation’s biomedical, behavioral, population, and clinical cancer research needs. The Florida-California Cancer Research, Education and Engagement Health Equity Center’s program focuses on training this underrepresented workforce. Our center is a tri-institutional collaboration between Florida A&M University, University of Florida, and University of Southern California. Here, we report the organizational structure and initial outcomes of our program to support 130 unique talented, underrepresented individuals in 138 cancer research training positions. Over the past 5 years, we offered the following: (1) a 12-week early and focused exposure of 35 undergraduate students; (2) training and support of 23 postbaccalaureate trainees in a 1-year mentored research and training program; and (3) academic career development, mentorship, and tailored research training opportunities to increase the competitive research capacity for 33 graduate students, 13 post-doctoral scholars, and 34 early-stage investigators. Educators, researchers, policymakers, and community leaders can use our training models to advance equity through excellence in education and research for underrepresented minority populations, ultimately fostering a more just and inclusive society.

## Introduction

Significant progress has been made over the past 30 years in reducing cancer morbidity and mortality in the United States (U.S.), with advancements in screening, diagnosis, and treatment [1]. However, despite these improvements, persistent racial and ethnic disparities continue to exist across the cancer care continuum. These disparities have far-reaching implications for underrepresented minority (URM) populations, particularly Black/African American (B/AA) and Hispanic/Latino (H/L) individuals.

B/AA individuals experience lower 5-year survival rates compared to their White counterparts across most cancer types, including breast, prostate, lung, and colorectal cancers [2]. Additionally, B/AA patients face higher cancer incidence and mortality rates, with shorter survival times even when controlling for socioeconomic factors. For example, the 5-year survival rate for breast cancer is significantly lower among B/AA women, despite the higher incidence of breast cancer screening in this population (American Cancer Society, 2022). Similarly, H/L individuals are often diagnosed at more advanced stages of cancer, particularly among first-generation immigrants, and they face growing cancer risks as their length of residence in the U.S. increases [3]. Cancer has become the leading cause of death among U.S.-born H/L individuals, surpassing heart disease [3].

The drivers of these disparities are multifactorial, encompassing socioeconomic status, healthcare access, insurance coverage, structural racism, and health literacy. Racial and ethnic minorities are less likely to have access to high-quality care, preventive services, and timely diagnosis, leading to worse outcomes. In addition to the physical and emotional toll on patients and their families, these disparities represent a significant economic burden. It is estimated that disparities in cancer care cost the U.S. healthcare system billions of dollars each year, compounding the costs associated with premature mortality and the use of emergency care services [4].

Addressing cancer disparities requires a multifaceted approach that includes focused research on URM populations. Increasing the representation of URM researchers in cancer research inclusion is one strategy that has been advocated by government agencies and professional medical societies to ensure that research is inclusive and reflective of the needs of all communities. Historically underrepresented groups must be involved in biomedical research at all levels to ensure that diverse perspectives and ideas are included in the scientific dialogue. This diversification is particularly critical for cancer health disparities research, where insights from researchers who are deeply connected to affected communities can lead to more effective and culturally relevant interventions.

The Florida-California Cancer Research, Education, and Engagement (CaRE^2^) Health Equity Center, funded by the National Cancer Institute (NCI), was established as a response to these challenges. The center represents a bicoastal partnership between the Florida Agricultural & Mechanical University (FAMU), the University of Florida (UF), and the University of Southern California (USC), with the goal of addressing cancer health disparities in B/AA and H/L populations. In this article, we present the organizational structure and initial outcomes of the CaRE^2^ partnership, with a particular focus on its Research Education Core (REC). The REC is dedicated to increasing the number of individuals from URM backgrounds pursuing careers in cancer research, by supporting and empowering them to contribute to eliminating health disparities in minority communities.

## Methods

### CaRE^2^ REC Overview

**Fig. 1:**
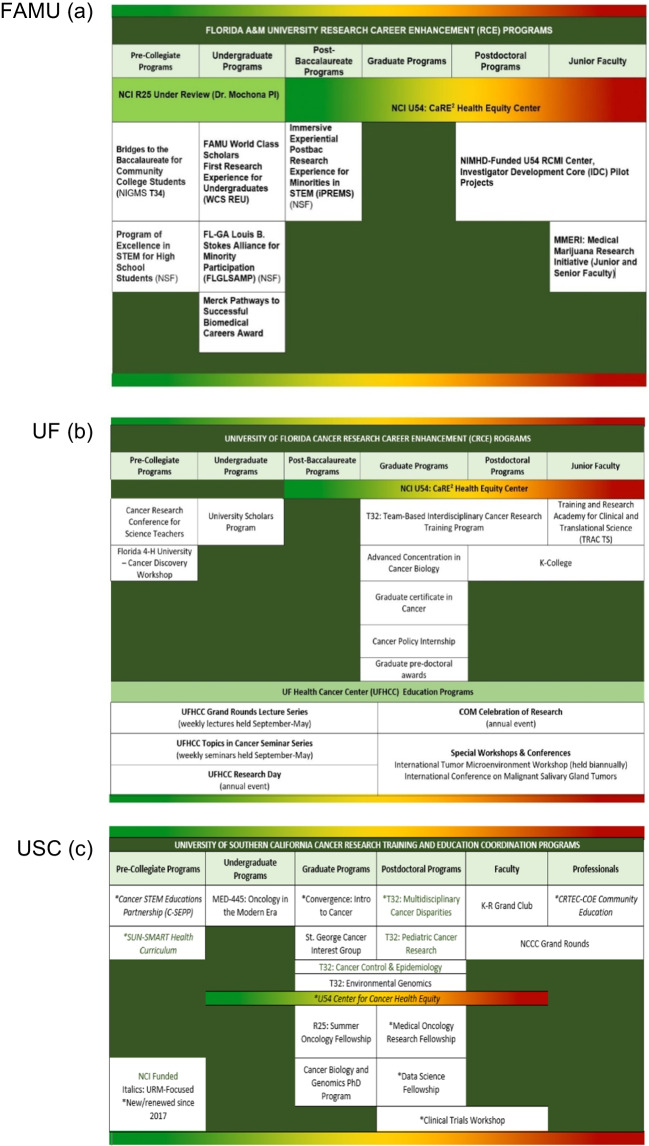
Other research career enhancement programs offered at FAMU (**a**), UF (**b**), and USC (**c**)

The CaRE^2^ Research Education Core (REC) is designed to increase the representation of URM scientists in cancer research by providing comprehensive, culturally sensitive training and professional development opportunities spanning multiple career stages. The program builds on the research education infrastructure of the three partner institutions—FAMU, UF, and USC—offering trainees access to a wide range of research technologies, mentorship, and career development resources (Fig. 1). The goal of the REC is to develop a pipeline of URM trainees who are well prepared to pursue careers in cancer health disparities research and who can contribute to addressing the unique challenges faced by underserved populations.

CaRE^2^ REC recognizes that the pipeline to careers in biomedical research often breaks early, with many URM undergraduate students not pursuing PhD or MD/PhD degrees and not continuing into academic careers. This progression gap is due to multiple factors, including a lack of adequate mentoring, role models, and institutional support [5]. CaRE^2^ REC specifically targets these barriers by focusing on the recruitment, retention, and long-term support of URM trainees. The program spans multiple career levels, including undergraduates, post-baccalaureate students, graduate students, postdoctoral fellows, and early-stage investigators (ESIs).

CaRE^2^ REC also emphasizes hands-on research training, exposure to cutting-edge research technology, and opportunities for interdisciplinary collaboration. By immersing trainees in cancer research that is directly focused on health disparities, the program aims to equip them with the knowledge and skills necessary to make meaningful contributions to the field.

### Organizational Structure

**Fig. 2:**
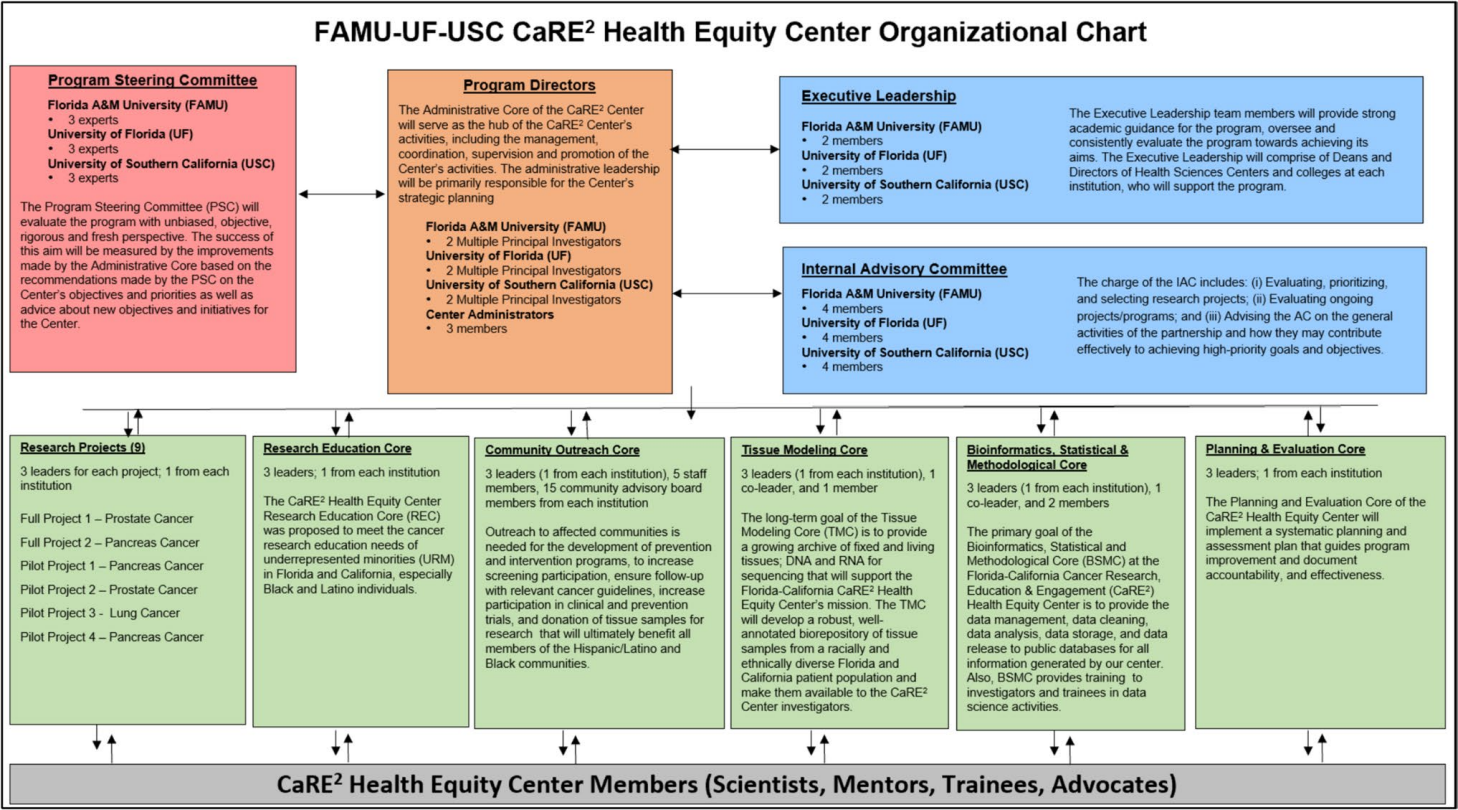
CaRE^2^ center organizational chart

The CaRE^2^ REC is part of the tri-institutional partnership between FAMU, UF, and USC (Fig. 2). Each institution plays a distinct role in the program, reflecting its strengths in training URM students. FAMU, the lead institution, is a historically Black college and university (HBCU) with a strong track record of preparing URM students for careers in biomedical research. FAMU has been recognized as one of the top producers of B/AA PhD graduates in the biomedical and behavioral sciences. The university’s nurturing academic environment, combined with its commitment to diversity, makes it an ideal partner in this initiative.

UF is one of the nation’s top public research universities, with a diverse student body and a strong focus on interdisciplinary research. The university’s six health-related colleges, including medicine, pharmacy, and public health, are located on a single, contiguous campus, facilitating cross-disciplinary collaboration. UF also houses the UF Health Cancer Center, which is an NCI-designated cancer center. USC, located in Los Angeles, provides access to one of the most diverse populations in the U.S., with students from various racial and ethnic backgrounds. USC’s Keck School of Medicine and Norris Comprehensive Cancer Center are well-known for their research on cancer disparities, particularly in H/L populations.

The organizational structure of the REC allows for shared leadership and decision-making among the three institutions. Leadership responsibilities rotate every 4 months, ensuring that each institution has an equal voice in the program’s operations. This collaborative structure fosters strong partnerships between the institutions and ensures that the needs of the trainees are met. Mentorship is a cornerstone of the REC, with each trainee paired with a mentor who guides them through the research process, helps them navigate their academic and professional development, and supports them in applying for research funding.

### Training Programs

The CaRE^2^ REC offers three primary training programs that span multiple career levels:

- 1. **Summer-CaRE**^**2**^ is a summer research internship program that provides undergraduate students from FAMU with hands-on experience in cancer health disparities research. The program offers a comprehensive introduction to research methodologies, data analysis, and academic writing, while also exposing students to advanced research technologies. In addition to laboratory research and mentorship, the program was intentionally designed to support students’ transition into graduate and professional schools. Structured components included workshops on writing personal statements, selecting appropriate programs, interview preparation, and guidance on obtaining letters of recommendation. Students also received individualized advising and near-peer mentoring to help them navigate the graduate or medical school application process. These design features, previously detailed by Mochona et al. [5], aimed to enhance the readiness and competitiveness of participants for advanced training opportunities.
- 2. **Postbac-CaRE**^**2**^ is a 1-year research education and career-development program designed for post-baccalaureate students who have not yet been admitted to graduate programs or medical schools. The program helps participants build strong research foundations by matching them with mentors who have active research portfolios. Trainees also have opportunities to present their research, engage in community outreach, and participate in service-learning experiences that allow them to understand how cancer disparities impact their communities.
- 3. **CaRE**^**2**^**-Grad + **is a research and career development program tailored for graduate students, postdoctoral fellows, and ESIs. The program focuses on building competitive research skills, providing mentorship in grant writing and publishing, and offering networking opportunities at national conferences. The program also encourages interdisciplinary collaboration, allowing trainees to work with researchers from across the CaRE^2^ partnership.

## Results

**Fig. 3:**
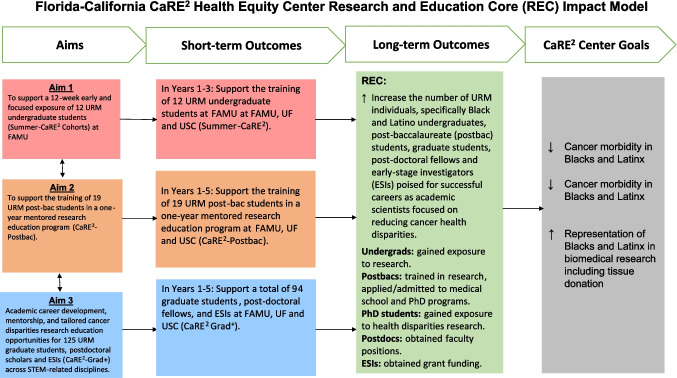
CaRE.^2^ research and education core impact model (2018–2023)

In its first 5 years as guided by the impact model displayed in Fig. 3, the CaRE^2^ REC trained a total of 130 unique URM trainees, exceeding its original training and career development benchmarks (Table 1). Notably, ten trainees transitioned to subsequent career stages and continued their training within the CaRE^2^ REC. Specifically, one trainee progressed from the Postbac-CaRE^2^ program into the CaRE^2^-Grad + program as a graduate student, five graduate student trainees advanced into postdoctoral training within CaRE^2^, and four postdoctoral trainees transitioned to early-stage investigator (ESI) roles while continuing with CaRE^2^ support. These internal transitions underscore the program’s capacity to sustain long-term engagement and support career progression across the academic pipeline.

**Table 1:** Benchmark goals and achieved for research education core trainees (2018–2023)

Metric	5-year goal	Achieved in first 5 years
Number of unique trainees	125	130 (126 URMs)
Scientific publications	39	313
Professional presentations	31	89
Grants submitted	29	> 53*
Grants awarded	10	53
Professional awards	5	22

*Precise figure unavailable

Over the course of our first 5 years, trainees participated in 138 career-level training positions, including undergraduate students, postbaccalaureate students, graduate students, postdoctoral fellows, and early-stage investigators. Of the 130 trainees, 126 (97%) were from URM backgrounds, reflecting the program’s commitment to fostering diversity in cancer research (Table 2).

**Table 2:** Demographic characteristics of the CaRE^2^ trainees and ESIs by career level positions* (2018–2023)

Variable	Overall (*n* = 138*)	UG (*n* = 35, 25%)	Postbac (*n* = 23, 17%)	Grad (*n* = 33, 24%)	Postdoc (*n* = 13, 9%)	ESI (*n* = 34, 25%)
Gender						
Male	49	**7**	8	14	5	15
Female	89	**28**	15	19	8	19
Race						
Black/African American	100	**34**	18	17	10	21
Native American/American Indian	6	**0**	0	2	0	4
Asian	4	**1**	0	1	0	2
Ethnicity						
Hispanic/Latino	31	**0**	6	14	3	8
Non-Hispanic/Latino	104	**35**	17	16	10	26
Partner institution						
FAMU	51	**31**	2	12	0	6
UF	41	4**	13	3	6	15
USC	46	**0**	8	18	7	13

Race and ethnicity do not equal overall total because several individuals identified as more than one race and ethnicity

*UG* undergraduate student, *Postbac* postbaccalaureate trainee, *Grad* graduate student, *Postdoc* postdoctoral trainee, *ESI* early-stage investigator

*Total number of 138 is career level training positions, not unique individuals

**Training program at FAMU with research mentorship at UF

### Summer-CaRE^2^ Program

The Summer-CaRE^2^ program trained 35 undergraduate students, nearly tripling the original target of 12 students. This success was achieved despite significant challenges posed by the COVID-19 pandemic, which disrupted academic and research activities across the U.S. The program’s focus on cancer health disparities resonated strongly with participants, many of whom expressed a desire to pursue careers that address these inequities. Of the 35 students, 31 have graduated, and 17 (47%) have applied for graduate or professional programs, including medical school and PhD programs. Nineteen students (54%) have been admitted to advanced programs, with many continuing their work in health disparities research. All 35 students presented their research at the annual CaRE^2^ symposium, where their abstracts were published in the symposium proceedings (Table 3).

**Table 3:** Expected outcomes for each training program

CaRE^2^ REC training program	Metric	5-year goal	Achieve by 5 years
Summer-CaRE^2^ *N* = 35	Application to medical school/graduate school	> 50%	49%*
	Admission to medical school/graduate school	> 50%	54%
	Presentation at national/regional meetings	100%	100%
Postbac-CaRE^2^ *N* = 23	Apply to graduate/medical school with an oncology-centered interest	> 50%	70%
	Admission to graduate/medical school	> 50%	65%
	Presentation of an abstract/poster at a national/international meeting	100%	96%
	Contribute to manuscripts each year as primary or co-author	50%	83%
	Submit a grant proposal (NIH/NCI research supplements grant or NIH/NCI individual predoctoral fellowship award F31	100%	0%
CaRE^2^- Grad + *N* = 80	Manuscripts published annually in peer-reviewed journals	> 6 per year (30 total)	> 280
	Submitted competitive grant applications per year would be submitted focused on cancer health disparities, including individual Predoctoral and postdoctoral fellowship awards, career development awards, and independent research awards	> 2 per year (10 total)	> 53
	Career progression of all trainees to the next level of scientific and academic career progression (e.g., to PhD or postdoctoral fellowships, tenure-track and tenured) paths focused on reducing cancer health disparities	100% (during the grant life cycle)	100%

For the summer CaRE^2^ program, 83% applied to medical/graduate school or are working in the health profession workforce

### Postbac-CaRE^2^ Program

The Postbac-CaRE^2^ program provided 23 postbaccalaureate students with a 1-year research experience and mentorship. The program’s emphasis on hands-on research and academic development helped trainees strengthen their applications for graduate and medical school. To date, 15 trainees have been admitted to advanced programs, including PhD and medical programs. The remaining students are in the process of applying. A majority (96%) of the trainees presented their research at national conferences, and 83% contributed as co-authors to published research articles. This level of research dissemination is a testament to the program’s success in preparing trainees for academic careers (Table 3).

However, the 1-year duration of the Postbac-CaRE^2^ program posed challenges for some trainees in developing robust preliminary data for competitive F31 applications. To address this, the REC has introduced additional support for grant writing earlier in the program timeline, along with structured mentorship to help trainees develop research proposals and strengthen their applications for external funding. The integration of these enhancements is expected to improve the success rates of future cohorts in securing competitive fellowships.

### CaRE^2^-Grad + Program

The CaRE^2^-Grad + program has been instrumental in advancing the careers of graduate students, postdoctoral fellows, and early-stage investigators (ESIs). Over the 5-year period, the program supported 80 trainees, including 33 graduate students, 13 postdoctoral fellows, and 34 ESIs. Grad + trainees published over 280 research articles, many of which focused on cancer health disparities (Table 3). One third (*n* = 26) of the Grad + trainees secured a total of 53 grants as principal investigators (Table 4), underscoring the program’s success in building research capacity. Notably, four URM ESIs were awarded R01 grants, which are highly competitive and represent a major milestone in establishing research independence.

**Table 4:** Grant award types by CaRE^2^ trainee type, 2018–2023

Award type	ESI	Trainee	Funders/funding mechanism
Federal (R01 equivalent)	8	–-	NIH (× 6), DOD (× 2)
Federal (other research grant)	6	2	R21 (× 3), R16, L60, P30, USAMRAA
Federal (*K* award/fellowship)	5	3	F30, F31, TL1, F99-K00, K01 (× 2), K23, K08
Federal (supplement)	2	2	NCI (× 3), NIH (× 1)
State	5	–-	FHD, TRDRP (× 4)
Foundation	15	–-	BMSF, ACS, OL, AF, ASTS. AASLDF
Internal awards	3	2	
**Total**	**44**	**9**	

*ACS* American Cancer Society, *ASTS* American Society of Transplant Surgeons, *DOD* US Department of Defense, *FHD* Florida Health Department, *NIH* National Institutes of Health, *NCI* National Cancer Institute, *TRDRP* Tobacco-Related Disease Research Program, *USAMRAA* US Army Medical Research Acquisition Activity, *FBCF* Florida Breast Cancer Foundation, *OL* OneLegacy, *AASLDF* American Association for the Study of Liver Diseases Foundation

In addition, seven postdoctoral trainees transitioned into faculty positions, demonstrating the program’s ability to support career progression. These accomplishments highlight the importance of long-term mentorship and the provision of opportunities for trainees to engage in collaborative research across institutions. The cross-institutional collaborations fostered by the CaRE^2^ partnership have enabled trainees to gain exposure to diverse research environments, enhancing their research capabilities and broadening their professional networks.

## Discussion

The CaRE^2^ REC has demonstrated significant success in preparing URM individuals for careers in cancer research, particularly in the field of cancer health disparities. By leveraging the strengths of three distinct institutions—FAMU, UF, and USC—the program has created a comprehensive and supportive environment for trainees to develop their research skills, engage in hands-on cancer research, and receive mentorship from established investigators. The program’s focus on fostering diversity in cancer research is aligned with national efforts to increase the representation of URM researchers and address health disparities in cancer care.

A key strength of the program is its ability to provide a continuum of research training that spans multiple career stages, from undergraduate students to early-stage investigators. This approach ensures that trainees receive sustained support as they progress through their academic and professional careers. The program’s emphasis on mentorship has been critical in helping trainees navigate the complexities of cancer research, secure external funding, and disseminate their findings through publications and presentations.

One of the most impactful aspects of the program has been its ability to motivate trainees by connecting their research to the communities most affected by cancer health disparities. Many trainees expressed a deep commitment to addressing health inequities in their future work, citing the program as a major influence on their career trajectory. The inclusion of community outreach and service-learning experiences has further strengthened this connection, allowing trainees to see firsthand the real-world implications of their research.

However, challenges remain. The relatively short duration of the Postbac-CaRE^2^ program limited some trainees’ ability to develop competitive fellowship applications, particularly for the NIH F31 grant. The introduction of earlier grant-writing support and the Cancer Health Disparities Research Academy are important steps in addressing this issue. These initiatives are expected to enhance the program’s ability to help trainees secure independent funding and advance their research careers.

Moving forward, the CaRE^2^ REC aims to build on its successes by expanding its initiatives and addressing areas for improvement. The Cancer Health Disparities Research Academy, which focuses on grant-writing training and career development, will be a central component of this expansion. The Academy is designed to provide structured support for trainees as they develop research proposals, submit grant applications, and build the skills needed to secure independent funding.

In addition to enhancing grant-writing support, CaRE^2^ REC will place a greater emphasis on increasing minority participation in clinical trials. Racial and ethnic minorities are often underrepresented in clinical trials, which can limit the generalizability of research findings and contribute to ongoing disparities in cancer treatment outcomes. By engaging URM scientists in clinical trial research, CaRE^2^ REC aims to address this gap and ensure that future cancer treatments are effective across diverse populations.

The program also plans to deepen its collaboration with community stakeholders to ensure that cancer research is not only scientifically rigorous but also culturally relevant. By involving community advocates and organizations in the research process, CaRE^2^ REC seeks to develop interventions that are tailored to the unique needs of underserved populations. This community-driven approach is critical for reducing cancer disparities and improving health outcomes in B/AA and H/L communities.

Furthermore, the CaRE^2^ partnership will continue to focus on recruiting and retaining URM trainees at all career levels. This effort includes expanding outreach tactics to attract a diverse pool of applicants and providing additional resources to support the retention of trainees throughout their academic and professional journeys. The program’s long-term goal is to create a sustainable pipeline of URM cancer researchers who are equipped to lead innovative research projects, secure external funding, and contribute to reducing cancer health disparities on a national scale.

Sustaining a program like CaRE^2^ REC beyond grant funding requires strong institutional commitment, infrastructure, and alignment with institutional diversity and research priorities. Over the past 5 years, each partner institution has demonstrated support through dedicated faculty time, office space, administrative support, and integration of CaRE^2^ activities into existing training and health equity–related initiatives. Additionally, collaborative structures such as shared mentorship networks and access to core resources (e.g., biostatistics, training workshops) have helped embed the program within each institution’s broader academic mission. Looking ahead, we recognize that continued institutional investment, along with diversified funding streams (e.g., additional NIH mechanisms, philanthropic support), will be critical to ensure long-term sustainability and continued progress toward diversifying the cancer research workforce.

## Conclusion

The CaRE^2^ REC training program has proven to be an effective model for preparing underrepresented minority trainees for careers in cancer research, with a particular emphasis on addressing cancer health disparities. By fostering a well-trained, culturally competent research workforce, the program has made significant strides in reducing inequities in cancer care and improving health outcomes for B/AA and H/L populations.

Through its innovative training programs, long-term mentorship, and commitment to diversity, the CaRE^2^ REC has provided trainees with the tools and resources needed to succeed in cancer research. The program’s success is evident in the high rates of trainee advancement to graduate and professional programs, the significant number of research publications and grants secured, and the transition of postdoctoral fellows into faculty positions.

As the program enters its next phase, CaRE^2^ REC will continue to expand its efforts to train the next generation of URM cancer researchers. By focusing on minority participation in clinical trials, strengthening community partnerships, and enhancing grant-writing support, the program is poised to make a lasting impact on cancer research and health equity. Ultimately, CaRE^2^ REC’s contributions will help to ensure that cancer research is more inclusive, culturally responsive, and better equipped to meet the needs of all populations.
